# Prevalence of Therapeutic use of Opioids in Chronic non-Cancer Pain Patients and Associated Factors: A Systematic Review and Meta-Analysis

**DOI:** 10.3389/fphar.2020.564412

**Published:** 2020-11-18

**Authors:** Helena De Sola, María Dueñas, Alejandro Salazar, Patricia Ortega-Jiménez, Inmaculada Failde

**Affiliations:** ^1^ The Observatory of Pain, University of Cádiz, Cádiz, Spain; ^2^ Preventive Medicine and Public Health Area, University of Cádiz, Cádiz, Spain; ^3^ Biomedical Research and Innovation Institute of Cádiz (INiBICA), Research Unit, Puerta del Mar University Hospital, University of Cádiz, Cádiz, Spain; ^4^ Department of Statistics and Operational Research, University of Cádiz, Cádiz, Spain

**Keywords:** chronic pain, meta-analysis, opioids, prevalence, systematic review

## Abstract

**Objectives:** To determine the prevalence and factors associated with the use of opioids among patients with chronic non-cancer pain (CNCP).

**Methods:** A systematic review and meta-analysis. Comprehensive literature searches in Medline-PubMed, Embase and SCOPUS databases. Original studies published between 2009 and 2019 with a cross-sectional design were included. The quality of the studies was assessed with Critical Appraisal Checklist for Studies Reporting Prevalence Data from the Joanna Briggs Institute. Protocol registered in the International Prospective Register of Systematic Reviews with reference number: CRD42019137990.

**Results:** Out of the 1,310 potential studies found, 25 studies fulfilled the inclusion criteria. Most of the studies were of high quality. High levels of heterogeneity were found in the studies included. In the general population, the prevalence of long-term opioid use was 2.3% (95% CI: 1.5–3.6%), the prevalence of short-term opioid use was 8.1% (95% CI: 5.6–11.6%), and among people with chronic low back pain it was 5.8% (95% CI: 0.5–45.5%). The prevalence of opioid use among patients from the health records or medical surveys was 41% (95% CI: 23.3–61.3%). Finally, in patients with musculoskeletal pain, the prevalence was 20.5% (95% CI: 12.9–30.9%) and in patients with fibromyalgia, 24.5% (95% CI: 22.9–26.2%). A higher prevalence of opioid use was observed among men, younger people, patients receiving prescriptions of different types of drugs, smokers and patients without insurance or with noncommercial insurance. In addition, non-white and Asian patients were less likely to receive opioids than non-Hispanic white patients.

**Conclusions:** The prevalence of opioid use among patients with CNCP was higher in subjects with short or occasional use compared to those with long-term use. Men, younger people, more chronic pain conditions, and patients without insurance or with noncommercial insurance were most related to opioid use. However, non-white and Asian patients, and those treated by a physician trained in complementary medicine were less likely to use opioids.

## Introduction

Chronic pain (CP) is a major public health concern (Vos et al., 2012) that is associated with disability, distress, and a decrease in the quality of life of affected individuals (de Sola et al., 2016). The prevalence of moderate to severe CP in the general adult population ranges from 2 to 55% in different countries (Azevedo et al., 2012; Mohamed Zaki and Hairi, 2015; Mills et al., 2019), with an estimated global annual cost of over US$245 billion (GSK, 2017).

The physiopathology of CP has been recognized to involve complex interactions between physical, psychological, and social factors, and that its appropriate management requires a multidisciplinary approach (Broekmans et al., 2010). However, pharmacological therapy remains a mainstay for treating these patients (Timmerman et al., 2016), opioids being one class of pharmacotherapies that is frequently prescribed to modulate pain (Parsells Kelly et al., 2008).

Opioid therapy has attracted growing interest recently related to the increased use observed in CP patients (Cheung et al., 2014; Alam and Juurlink, 2015; Webster et al., 2017). This situation is of particular concern in patients with chronic non-cancer pain (CNCP), where the evidence of opioid therapy benefits may be less robust than that observed in patients with acute or cancer pain (Von Korff et al., 2011; Scholten, 2013; Campbell et al., 2015). The duration of opioid therapy is also important with regard to the benefits for patients, since the prescription of opioids may be appropriate for short-term pain relief, but long-term opioid therapy (LTOT) cannot be associated with improvements in pain or function (Karmali et al., 2020).

Furthermore, despite the lack of information on the efficacy of opioids (Warner, 2012), the introduction of high-dose and extended-release oral tablet formulations of opioids has been shown to increase the total prescriptions among CNCP patients, especially in the last decade (Von Korff et al., 2011; Alam and Juurlink, 2015; Severino et al., 2018). In some European countries, such as Spain, the use of opioids increased by 83.59% from 2008 to 2015 (De Sola et al., 2020). Additionally, in 2016, more than one-third of adults were prescribed opioids in the United States (Walker, 2018), making it an important social problem (Sehgal et al., 2012; Salazar et al., 2019). The differences in opioid prescribing patterns have been related to age, gender, ethnicity, pain diagnosis, number of total medications, payment type, physician specialty, and patient relationship with provider (Rasu and Knell, 2018).

Determining the prevalence of their use and factors underlying its prevalence can advance our understanding of current treatment practice and its impact on public health. Thus, it is necessary to collect updated information about the prevalence of the therapeutic use of opioids for CNCP in different countries, and summarize the information published. Additionally, it is necessary to take into account the length of the treatment and factors associated with it to produce international estimates.

To this end, we carried out a systematic review of the literature to know the prevalence of the therapeutic use of opioids in patients with CNCP and, as a second aim, to analyze the factors associated with their use. We also performed a meta-analysis of the prevalence of the therapeutic use of opioids to summarize the information obtained.

Following the PICOS method, the research question of this systematic review is: What are the prevalence and factors associated with the use of opioids among patients with CNCP?

## Methods

### Protocol and Registration

The present systematic review and meta-analysis was conducted following the Preferred Reporting Items for Systematic Reviews and Meta-Analysis statement (Shamseer et al., 2015) (Supplementary Material SI). The study protocol was registered in the International Prospective Register of Systematic Reviews with reference number: CRD42019137990.

### Design of the Study

Systematic review and meta-analysis.

### Search Strategy

A systematic search strategy was built according to PICOS method and performed in the Medline-PubMed, Embase and SCOPUS databases. The terms/keywords of interest were “opioid,” “analgesic,” and “pain.” The terms were combined with the tag for searching in title, abstract and keywords. Search terms and search strategies were adapted to each database (Supplementary Material SII, TI). In light of the differences in the prevalence of opioid use in the last decade, the recent original studies, i.e., published in English or Spanish from January 2009 to December 2019 with a cross-sectional design were included.

Once the search strategies for all the databases were executed, we imported all the references found into the Covidence online tool (Veritas Health Innovation Ltd., 2019). The process of duplicate removal, screening, data extraction and risk of bias analysis were performed by this web-based systematic review tool.

### Eligibility Criteria

The population of interest was people (all ages) with chronic non-malignant pain. Those studies related to CNCP located in specific body regions (e.g. musculoskeletal CP) were also included. Thus, the term “CNCP” was not included in the search strategy in order not to limit the searches to studies presenting only data from general CNCP. The criteria to define CNCP and the specific body regions that each study focuses on are specified in Table 2.

In this review, we exclusively focused on opioid treatment as an intervention for pain. The use of opioid treatment can be defined as self-reported use, self-reported prescription medications and prescription, or dispensed drugs retrieved from electronic health records (see Table 2). A study was selected when its main outcome was the prevalence of the use of opioids in CNCP, as long as these data were shown within the study or it was possible to calculate prevalence from it. Studies analyzing the factors associated with the use of opioids were also included.

Studies reporting the use of opioids in patients with acute, post-operative, palliative, or cancer pain were excluded. Studies focusing on the opinions or attitudes of physicians about opioid prescription or on the disorders derived from their consumption were also excluded (Supplementary Material SII, T2).

### Study Selection

Two authors (MD and HS) independently screened the title and abstract of all of studies. Shortlisted studies were then analyzed in depth according to the inclusion criteria, and their reference lists were also revised to identify studies that could be included in the review.

### Risk of Bias Assessment

The quality of the studies was assessed following the Critical Appraisal Checklist for Studies Reporting Prevalence Data from the Joanna Briggs Institute (Munn et al., 2015). This checklist consists of nine items regarding sample frame, appropriate recruitment, adequate sample size, appropriate description of the subjects and setting, data analysis, method used, the reliability of condition measures, appropriateness of the statistical analysis and response rate (Table 1). Each item was assessed as “yes,” “no,” “unclear” or “not applicable.” For standardization, “yes” was considered to imply a low risk of bias, and “no” and “unclear” a high risk of bias. Since there is not a standard classification for the “Critical Appraisal Checklist for Studies Reporting Prevalence Data from the Joanna Briggs Institute,” some systematic reviews were consulted to guide our classification (Porto De Toledo et al., 2017; Bett et al., 2019; Peterson et al., 2019). A study was considered to have a low risk of bias (i.e., high-quality study) when it accumulated at least seven items answered as “yes” and a moderate risk of bias when the study reached 4–6 “yes.” Any disagreements regarding the suitability of a study were resolved by a third author (AS).

**TABLE 1:** Risk of bias assessment. Checklist for studies reporting prevalence data from the Joanna Briggs Institute (N = 25).

Author name, year	Q1	Q2	Q3	Q4	Q5	Q6	Q7	Q8	Q9
Ahn, 2016	Yes	Yes	Unclear	Yes	Yes	Yes	Yes	Yes	NA
Azevedo, 2013	Yes	Yes	Yes	Yes	Yes	Yes	Yes	Yes	Yes
Birke, 2016	Yes	Yes	Yes	Yes	Yes	Yes	Yes	Yes	Yes
Callhof, 2019	Yes	Yes	Yes	Yes	Yes	Yes	Yes	Yes	Yes
Fain, 2017	Yes	Yes	Yes	Yes	Yes	Yes	Yes	Yes	NA
Fredheim, 2014	Yes	Yes	Yes	Yes	Yes	Yes	Yes	Yes	No
Gouveia, 2017	Yes	Yes	Yes	Yes	Yes	Yes	Yes	Yes	No
Häuser, 2012	Yes	Yes	Unclear	Yes	Yes	Yes	No	Yes	No
Henderson, 2013	Yes	Yes	Yes	Yes	Yes	Yes	Yes	Yes	Yes
Kingsbury, 2014	Yes	Yes	Yes	Yes	Yes	Yes	Yes	Yes	No
Knoop, 2017	Yes	Yes	Yes	Yes	Yes	Yes	Yes	Yes	No
Kurita, 2012	Yes	Yes	Yes	Yes	Yes	Yes	Yes	Yes	Yes
Larochelle, 2015	Yes	Yes	Yes	Yes	Yes	Yes	Yes	Yes	NA
Lin, 2019	Yes	Yes	Yes	Yes	Yes	Yes	Yes	Yes	NA
Marschall, 2015	Yes	Yes	Yes	Yes	Yes	Yes	Yes	Yes	NA
Miller, 2017	Yes	Yes	Yes	Yes	Yes	No	Yes	Yes	Yes
Miller, 2019	Yes	Yes	Yes	Yes	Yes	Yes	Yes	Yes	NA
Rodondi, 2019	Yes	No	Yes	Yes	Yes	No	Yes	Yes	Yes
Romanelli, 2017	Yes	Yes	Yes	Yes	Yes	No	Yes	Yes	NA
Scala, 2018	Yes	Yes	Unclear	Yes	Yes	No	Yes	Yes	No
Shmagel, 2018	Yes	Unclear	Yes	Yes	Yes	Yes	Yes	Yes	Unclear
Sites, 2018	Yes	Yes	Yes	Yes	Yes	Yes	Yes	Yes	NA
Van den Driest, 2019	Yes	Yes	Yes	Yes	Yes	Yes	Yes	Yes	Yes
Vincent, 2015	Yes	Yes	Yes	Yes	Yes	Yes	Yes	Yes	No
Wand, 2016	No	No	Yes	Yes	Yes	Yes	Yes	Yes	Unclear

Q1, Was the sample frame appropriate? Q2, Participants were appropriately recruited? Q3, Sample size was adequate? Q4, Study subjects and setting were described? Q5, Was the data analysis conducted with sufficient coverage of the identified sample? Q6, Were valid methods used for the identification of the condition? Q7, Was the condition measured in a standard, reliable way for all participants? Q8, Was there appropriate statistical analysis? Q9, Was the response rate adequate or managed appropriately? NA, Not applicable.

### Data Extraction

Information was extracted about the primary aim of the study, the characteristics of the population, the sample source, sample size, method for data retrieval, response rate, the definition of CNCP considered in each study, the prevalence of CNCP in the population studied, the prevalence of opioid use, the method for obtaining this prevalence data, and the factors associated with opioid use (Table 2).

**TABLE 2:** Characteristics of the studies included in the systematic review.

First author, Year	Primary aim	Population	Sample source and timeframe	Method for data retrieval	N	Response Rate	Pain definition	Prevalence of CP	Method for obtaining the opioids prevalence	Prevalence of opioid use	FA to opioid use in CP patients
(Miller et al., 2019)	To analyze the prevalence of non-opioid prescribing among commercially- insured patients with CP	The commercially insured population (18–64 years)	MarketScan database. 2014	Truven health MarketScan Research	21,745,233	NA	Patients with at least two outpatient visits in 90 days for CP ICD-9-CM code	9.5%	Prescription drugs coverage using the National Drug code schema	28.4% had prescriptions for both opioids and a non-opioid, 15.9% prescription for an opioid	—
(Rodondi et al., 2019)	To investigate among PC patients and their physicians in western Switzerland the prevalence of use, perceived usefulness, and communication about treatments for CLBP including complementary medicine	Patients with CLBP recruited during regular medical appointment. (≥18 years)	PC physician in western French-speaking area of Switzerland from November 1, 2015, to May 31, 2016	Self-reported questionnaire	499	37%	CLBP, defined as pain lasting or recurring for 3 months or more	The whole sample had CLBP (N = 499)	Self-reported use of assessed therapies	52.5% of the CLBP patients	Current smoking was associated with using opioids (OR = 1.8; 95% CI: 1.1–3.1) - Patients from PC physician who were trained in complementary medicine were significantly less likely to use opioids (OR = 0.5; 95% CI: 0.3–0.9)
(Callhoff et al., 2020)	To analyze FA with the burden of OA, taking the pattern of joint involvement into account	Patients with OA of the knee or hip or with POA (30–79 years)	German statutory health insurance database (BARMER). Year 2016	Survey and claims data	8,995	42%	Persons with ICD- 10- GM (German Modification) diagnoses of OA in 2014	The whole sample had OA (N = 3,564) - 758 POA - 959 hip OA. - 399 hip and knee OA- 1,448 knee OA	Analgesics were identified using ATC codes, counting patients as users if they had ≥1 prescription of the drug in that year	14.9% (n = 531: 106 POA+134 hip OA+88 hip and knee+203 knee OA) of the total OA patients - 14% POA. - 14% hip OA. - 22% hip and knee OA- 14% knee OA.	—
(Lin et al., 2019)	To examine how prescription drug monitoring programs share data with bordering states and its association with patients being prescribed opioids for non-cancer CP treatment	Adult patients with CNCP (≥18 years)	National Ambulatory Medical Care Survey (NAMCS) 2014	The NAMCS	Weighted N = 66,198,751; unweighted N = 2,846	NA	The ICD-9-CM diagnosis codes provided by the NAMCS	The whole sample had CNCP (N = 2,846)	Electronic health records, including information on prescribed medications	33.1% of the study sample	- Patients aged 25–49 vs. 18–25 years (OR = 2.78; 95% CI: 0.93–8.33) - Patients with Medicare (OR = 1.56; 95% CI: 1.03–2.38) or Medicaid coverage (OR = 2.08; 95% CI: 1.15–3.85) vs. who had private insurance coverage. - Patients being followed by the physician vs. naïve patients (OR = 2.33; 95% CI: 1.49–3.57)
(Van Den Driest et al., 2019)	To examine the analgesic used by patients with OA related pain and how the analgesics were used in the preceding month	Patients with rheumatic diseases (age not specified)	The panel of the Dutch Arthritis Foundation	Online questionnaire	842	56%	Generalized OA was defined as self-reported OA in 3 or more groups of joints	The whole sample had OA (N = 842)	Self-reported medications used in the preceding month for OA related pain	22% (n = 186: 44 use only opioids +142 use opioids combined with others drugs) of the patients with OA-related pain	—
(Shmagel et al., 2018)	To examine patterns of drugs prescription among Americans with CLBP in a nationally representative, community-based sample	A representative sample of US adult population (aged 20–69)	The National health and Nutrition Examination Survey. 2009–2010	Home-based interviews with pill bottle verification to capture prescribed medications for CP.	5,103	NR	CLBP was defined as self-reported pain in the area between the lower posterior margin of the ribcage and the horizontal gluteal fold on most days for at least 3 months	13.7% had CLBP (N = 700)	Self-reported prescription medications used within the past 30 days	18.8% of working-age Americans with CLBP	- Low levels of education: For less than high school (OR = 3.07; 95% CI: 1.12–8.39) and for high school or associates’ degree (OR = 4.17; 95% CI: 1.73–10.03) compared with college education - <35,000$ of annual household income (OR = 1.92; 95% CI: 1.19–3.11) vs > 65,000 2 or more medical comorbidities (OR = 3.32; 95% CI: 1.74–6.35) vs none or one
(Scala et al., 2018)	To evaluate the level of readiness to practice different types of active self-care among CP patients	Patients with CP. (≥18 years)	Patients seeking care at the Pain Center University Hospital, Switzerland between June 2013 and March 2015	Self- administered questionnaire	639	41.9%	Pain lasting 6 months or more	The whole sample had CP (N = 639). The locations were back (71.4%), lower limb (68.4%), cervical spine (25.8%), an upper limb (25.2%) or a shoulder (23.0%)	Patients were asked whether they used non-opioid painkillers, opioids or dietary supplements ‘against pain” during the last six months	64.6% of the study sample	—
(Sites et al., 2018)	To understand the relationship between prescription opioid use and satisfaction with care among adults with musculoskeletal conditions	Patients with musculoskeletal conditions (≥18 years)	Nationally representative data from the 2008–2014 Medical Expenditure Panel Survey	5 rounds of telephone interviews over a 30-month period and questionnaire	19,566	NA	A combination of (ICD-9-CM) codes and patient self-reported data	The whole sample had musculoskeletal pain (N = 19,566)	Participants were asked to report prescription medication use and pharmacies were contacted to validate these prescriptions	13.1% opioid users. - 29.2% as low-level users (2–4 opioid prescriptions) - 28.9% as moderate users (5–9) - 41.9% as heavy users. (10 or more)	—
(Knoop et al., 2017)	To describe the use of analgesics; and to determine FA with analgesic use in patients with knee and/or hip OA referred to an outpatient center	Patients referred to an outpatient center with knee and/or hip OA diagnosed (age not specified)	Amsterdam Osteoarthritis (AMS-OA) cohort in an outpatient center (reade, center for rehabilitation and rheumatology, the Netherlands) from December 2009 to July 2016	Questionnaire	656	NR	Clinical knee and/or hip OA diagnosed, according to the American college of rheumatology criteria	The whole sample had OA (N = 656)	Patients were asked to list all medication used at that moment	12% use of opioids 6% Tramadol 3% codeine 1%Prednisone 3% Other	—
(Miller et al., 2017)	To estimate the prevalence of CP and analgesia use in the Australian population by: age and sex; the severity of pain in the population with CP by sex; and the distribution of recent pain severity in those using analgesia by age and sex	Representative sample of Australian population. (All ages)	The ABS	Face-to-face interviews conducted by trained ABS interviewers in participants' homes	n = 20,426 participants from 15,565 private residences. 1 adult and 1 child aged 0–17 years (if applicable) in each participating household	84.8%	Self-reported pain, which persisted over a 6-month period	- 12.7% of all ages (N = 2.8 million) - 15.4% (aged ≥15 years). -14.6% males -16.1% females	Opioid analgesia use included the any type of opioid analgesia over the previous 2 weeks. Participants were asked for the name or brand of all medication and to provide the packages to the interviewer	12% males vs. 13.4% females (aged ≥15 years)	—
(Romanelli et al., 2017)	To evaluate opioid prescribing in an ambulatory setting among patients with CNCP	Adult patients with CP with a medical record in the EHR (≥18 years)	Using Sutter EHR (community-based open-network healthcare system in northern California)	The EHR	1,784,114	NA	Patients with 2 records of ICD-9 CM, diagnoses for a CNCP condition (pain lasts longer 3 months) at least 30 days apart	6.8% (N = 120,481)	The electronic health records, including information on prescribed medications	Patients receiving any opioids among all CP Patients: 58.1% Short-acting (immediate-release) opioids: 57.4%. Long-acting opioid: 7%	CP Conditions per Patient by CP category: Arthritis/joint pain (OR = 1.39; 95% CI: 1.36–1.42) Back/cervical pain (OR = 1.07; 95% CI: 1.05–1.09) Neuropathies/neuralgias (OR = 1.65; 95% CI: 1.61–1.69) Headaches/migraines (OR = 1.51; 95% CI: 1.47–1.56), unclassified pain (OR = 1.48; 95% CI: 1.44–1.53). **Patient demographic characteristics** Older patients (≥66 years vs 18–45 years) (OR = 0.55; 95% CI: 0.52–0.58) Those with moderate chronic disease burden (CCI score = 2–3 vs 0) (OR = 0.92; 95% CI: 0.88–0.96) asians (vs. Non-Hispanic-White) (OR = 0.37; 95% CI: 0.33–0.40) **Patients with higher odds of receiving an opioid were:** Men (over women). Patients with non-commercial insurance, especially Medicaid (OR = 2.77; 95% CI: 2.56–3.01) Patients with more CP conditions (OR = 3.27; 95% CI: 3.15–3.40)
(Fain et al., 2017)	To quantify prescription analgesic use of elderly nursing home residents with persistent non-cancer pain and to identify individual and facility traits associated with no treatment	Elderly nursing home residents with persistent non-cancer pain. (≥65 years)	Individuals residing in a nursing home in U.S. at any time between December 2007, and November 2008	The Minimum Data Set; the Online Survey, certification, and Reporting database; and Medicare Part D	2.99 million individuals	NA	Moderate to severe daily pain lasting at least 3 months	3.8% (N = 18,526) of eligible nursing home residents had persistent pain	Prescription drug used from Medicare Part D records. An opioid prescription dated within 30 days before or after persistent pain onset.	- 81.2% received an opioid drug (alone or in combination with acetaminophen or prescription NSAID). -16.2% had only opioids prescription	—
(Gouveia et al., 2017)	To analyze and characterize the intake profile of pain-relief drugs in a population-based study of adults with CLBP.	Portuguese adult population With self-reported active CLBP (>18 years)	Households selected by random route methodology	Face-to-face interview	10,661	NR	LBP lasting at least 90 days	10.4% (CI 9.56%; 11.9%) (N = 1,487)	Information regarding analgesic and other pain-relief drugs was collected and organized according to the national drug agency classification	1.6% (95% CI: 0.9–2.2) among population with active CLBP	—
(Ahn et al., 2016)	To assess medical care and costs of the 3 highest prevalence lumbar disorders -nLBP, IDD and SS- to provide basic information for standards of appropriate management	Patients included in 2011 Korean health Insurance Review and assessment Service (HIRA) (all ages)	National health Insurance billing data provided by HIRA. Year 2011	2011 HIRA National Patient Sample (NPS)	1,375,842	NA	Patients with a lumbar disorder coded by the Korean Classification of Diseases, adapted from the ICD-10	27% (Patient visiting medical institution with lumbar/spinal diagnostic codes N = 371,858)	Treatment prescriptions classified according to National Evidence-based healthcare collaborating Agency reports	2.3% (n = 4,300: 761 nLBP +1994 IDD +1545 SS) of the total registers with lumbar disorder included for analyses (N = 188,985: 111,544 nLBP +48,413 IDD +28,842 SS)	—
(Birke et al., 2016)	To examine the trends regarding the prevalence of CNCP, dispensed opioids, and concurrent use of BZD/BZD-related drugs in the Danish population	Participants with CP (≥16 years)	The Danish National Cohort Study (DANCOS). Years 2000, 2005, 2010 and 2013	In 2000 and 2005, face-to-face interviews and self- administered questionnaire. In 2010 and 2013, postal or web questionnaire	16,684 in 2000 10,916 in 2005 25,000 in 2010 25,000 in 2013	63% in 2000 51% in 2005 61% in 2010 57% in 2013	Pain lasting 6 months or more	-18.9% in 2000 - 20.2% in 2005 - 26.2% in 2010 - 26.8% in 2013	Dispensed medicines from the Danish National Prescription registry using ATC codes. **Long-term,** having used at least one prescription/month for 6 months. **Short- term** having used at least one prescription in the previous year	Opioid users among individuals with CP Long-term - 1.3% in 2000. - 1.3% in 2005 - 1.7% in 2010. - 1.8% in 2013. Short-term - 2.8% in 2000. - 3.1% in 2005 - 3.8% in 2010. - 3.9% in 2013	—
(Wand et al., 2016)	To present the outcomes of a comprehensive evaluation of the psychometric properties of the Fremantle Back Awareness Questionnaire and explore the potential relationships between body perception, nociceptive sensitivity, distress, and beliefs about back pain and the contribution these factors might play in explaining pain and disability	People with axial CLBP (between 18 and 70 years)	From 2 metropolitan hospitals in Western Australia, private metropolitan physiotherapy clinics, pain management and general practice clinics. Also, via multimedia advertisements circulated throughout the general community Western Australia	Self- administered questionnaire and a combination of clinical bedside tests and laboratory tests	251	NR	To have experienced LBP for >3 months, scored ≥2 on a numeric rating scale, and ≥5 on the roland Morris Disability Questionnaire	The whole sample had experienced LBP	Self-reported questionnaire about current pain medications	15.9% of the 251 people with CLB	—
(Vincent et al., 2015)	To evaluate the problem of multiple chronic conditions and polypharmacy in patients with fibromyalgia	Patients with fibromyalgia. (≥21 years)	Patients identified via the rochester Epidemiology Project in Olmsted county, Minnesota. Between January 2005 and December 2009		1,111	NR	Patients with a diagnosis of fibromyalgia (HICDA or ICD-9)	The whole sample had fibromyalgia	Using a unit (or dossier) medical record system, whereby data from an individual (demographics, diagnoses and billing records)	22.4% among the 1,111 patients with fibromyalgia	—
(Larochelle et al., 2015)	To characterize trends in opioid prescribing and co-prescribing of sedative hypnotics at acute and chronic musculoskeletal pain visits from 2001 to 2010	Patients with musculoskeletal pain. (≥18 years)	Combining the NAMCS and National Hospital Ambulatory Medical Care Survey	Data collection was carried out by physicians, hospital staff, or census field representatives	35,302	NA	Pain lasting at least 3 months	53% of the visits were for CP.	Drugs prescribed using Multum drug classification. The primary outcome was prescription or continuation of an opioid medication during the visit	Combining all years, opioids were prescribed to 20.8% (95% CI 18.9–22.6%) of CP visits. 12.9% (95% CI 9.7–16.0%) in 2001.28.2% (95% CI 21.4–34.9%) in 2007.23.1% (95% CI 18.3–27.9%) in 2010	Patients aged 35–49 years vs. 50–64 years (OR = 1.32; 95% CI: 1.11–1.56). Hispanic vs. non-Hispanic whites (OR = 0.54; 95% CI: 0.39–0.74). Patients with Medicaid (OR = 1.46; 95% CI: 1.16–1.85), Medicare patients under age 65 years (OR = 2.34; 95% CI: 1.77–3.10), and patients without insurance (OR = 1.54; 95% CI: 1.21–1.96) vs. private insurance. Patients visiting their assigned PC provider (OR = 1.39; 95% CI: 1.15–1.68) and patients previously seen in that office (OR = 1.94; 95% CI: 1.52–2.49)
(Marschall et al., 2015)	To determine the prevalence and the demographic and medical predictors of LTOT, of high doses of LTOT and of abuse/addiction of prescribed opioids in a cohort of insureds with CNCP of a large German statutory health insurance	Persons insured by the German statutory medical health insurance. (Age not specified)	From the records of outpatient (Association of Statutory health Insurance Physicians bills) and inpatient care (hospital bills) of persons, insured by the German statutory medical health insurance plan Barmer GEK January 2012 and December 2012	The Barmer GEK.	870,000	NA	According to the ICD-10-GM	The whole sample had CP.	Oral opioid prescriptions of outpatient care were identified by the Anatomical Therapeutic chemical Classification (ATC). The insurance organization **LTOT prescriptions**: Defined by at least one opioid prescription per quarter for at least three consecutive quarters (one quarter = 3 months) over the last 12 months. **High-dose** opioid therapy (defined by ≥ 100 mg MEQ/day)	LTOT prescription all insureds with CNCP 1.3% (range 1.2%; 1.4%). High-dose opioid therapy among LTOT patients 15.5% (range 14.2%; 16.5%)	—
(Kingsbury et al., 2014)	To examine the impact of peripheral joint OA across five large European countries and how people with OA use pharmacotherapies	The general population using the internet panel maintained by Lightspeed Research. (≥18 years)	Data were derived from the 2011 five European countries (5 EU) National health and Wellness Survey (NHWS)	Respondents were emailed a link to the survey to complete on their own. ≥65-year-old population were recruited by telephone and they had the choice to complete the interview on the phone	57, 512: France: n = 15,000 Germany:n = 15,001 Italy: n = 7,500 Spain: n = 5,011 UK: n = 15,000	NR	Respondents who self-reported a physician diagnosis of OA	OA prevalence 6.5% - UK 10.9% - France 6.4% - Germany 3.8% - Spain 6.3% - Italy 3.6%	Respondents were asked whether they currently use prescription to treat their arthritis; if so, they were asked to indicate what they were currently using	16.7% among respondents with diagnosis of OA (N = 3,750) - 19.3% in the UK (N = 1,635) - 27.7% in France (N = 961) -3.5% in Germany (N = 570) - 6.9% in Spain (N = 316) - 0.7% in Italy (N = 268)	—
(Fredheim et al., 2014)	To know the prevalence of persistent opioid use among people in the general population with self-reported CNCP	All inhabitants in the county of Nord-Trondelag in Norway (≥20 years)	Linkage of the National Norwegian prescription database and the Nord-Trøndelag health study 3 2006–2008	2 Postal questionnaires and a physical examination	45,837	NR	Pain lasting 6 months or more and pain of at least moderate intensity during the last week before participation in HUNT 3	31.6%	Prescription drugs dispensed at pharmacies from The National Norwegian Prescription Database. Two different definitions of **persistent opioid use** included: The wide definition clinically corresponds to using opioids most days of the week (>180 DDD or 4500 OMEQ) -The strict definition to using opioids around the clock all days (>730 DDD or 18,000 OMEQ). Data on dispensed opioid prescriptions during the 6 months immediately before participation in HUNT 3	Opioid users among individuals with CP Persistent opioid use 2.9% Occasional opioid use 12.3%	- Being younger than 56 years old (OR = 2.22; 95% CI: 1.65; 2.99) - Male (OR = 1.49; 95% CI: 2–1.11) - A current smoker (OR = 2.95; 95% CI: 1.36–2.94) - using more than 100 DDD of benzodiazepines per year (OR = 5.55; 95% CI: 3.74–8.23) - Receiving prescriptions of drugs from several ATC classes (OR = 4.98; 95% CI: 3.31–7.48)
(Azevedo et al., 2013)	To describe the prevalence and FA with opioid use in subjects with CP in Portugal and to evaluate satisfaction and self-assessed treatment effectiveness	A representative sample of the adult Portuguese population (≥18 years)	Random digit dialing	A structured questionnaire conducted by CATI.	5,094	76% among responding households and 51% among all identified households	Pain lasting at least 3 months	35.7% (95% CI: 34.38–37.02)	Respondents were asked if they were using any pain medicine. If so, they were asked for the drugs and the frequency	4.24% (95% CI: 3.31–5.41) among participants who responded if they were using any pain medicine (N = 1786)	-Pain-related disability PDI (per increase in 10 units) (OR = 1.23; 95% CI: 1.02–1.50)
(Henderson et al., 2013)	To determine the prevalence of CP, its causes, severity, management, impact on sleep, mood and activity levels, and GP and patient satisfaction with pain management	Patients attending General Practice. (All ages)	The BEACH (Bettering the Evaluation and Care of health), an Australian General Practice program	Questionnaires were completed by the GP in discussion with the patient, using the combined knowledge of both	5,793	79%	Pain experienced every day for three months in the six months prior to this consultation	18.8% (95% CI: 17.8–19.8)	Respondents were asked if their pain was being managed and how. If the answer was “with medication”, they were asked to specify which medication	32.7% (n = 343: 365 took opioids - 22 people with cancer) among respondents with CNCP who responded to the management question (N = 1,049 =1,074 – 25 people with cancer)	—
(Häuser et al., 2012)	To conduct the first European FMS consumer reports on the effectiveness and side effects of FMS-therapies in routine clinical care	Members of the self-help organizations with diagnosis of FMS (age not specified)	From the two largest German FMS-self-help organizations and nine clinical institutions. 2010–2011	Self-reported questionnaires	1,661	NR	FMS-diagnosis >1 month’s duration	The whole sample had FMS	Participants were asked to “indicate whether they currently use any interventions for FMS”. The interventions, including drugs, were listed in different sections	- 17.6% Weak opioids - 8.4% Strong opioids	—
(Kurita et al., 2012)	To estimate the current prevalence of CP in the Danish population, CP prevalence in immigrants of different origin in Denmark, CP prevalence related to potential FA.	Individuals living in Denmark (≥16 years)	From the Danish health Survey and official Danish health and socioeconomic, individual-based registers. 2010	A self-reported survey questionnaire and administrative registers	25,000	60.7%	Chronic Pain: Participants who responded they had chronic/long-lasting pain lasting 6 months or more	24.7% (95% CI: 24.0–25.4) CNCP (N = 14,925)	Dispensed prescription medications obtained from the Danish National Prescription registry and linked on an individual level to the survey data. Use of medication was defined as at least 1 dispensed drug during the 90 days before survey completion	12.3% (N = 3,305)	—

EHR, Electronic health Record system; ABS, Australian Bureau of Statistics; NAMCS, National Ambulatory Medical Care Survey; POA, polyarthritis; FMS, fibromyalgia syndrome; LTOT, long-term opioid therapy; CP, chronic pain; PC, primary care, CLBP, chronic low back pain; LBP, low back pain; nLBP, non-specific low back pain; IDD, intervertebral disc disorder; SS, spinal stenosis; CNCP, chronic non-cancer pain; OA, osteoarthritis; BDZ, benzodiazepine; FA, factors associated; GP, general practitioner; CATI, computer-assisted telephone interviews; NA, not applicable; NR, not reported (Mezcla de mayúsculas y minúsculas).

### Statistical Analysis

A descriptive analysis of the characteristics of all the studies included in the systematic review was carried out. A meta-analysis was performed if two or more studies reported the same characteristics in the information provided and the same measure of effect. In order to manage heterogeneity, the studies were first grouped according to the following aspects: the source of the sample (the general population or health registries/medical surveys); the duration of opioid treatment [long-term, commonly defined as more 3 months or short-term (Karmali et al., 2020)] and the type of pain (Table 3).

**TABLE 3:** Characteristics of the subgroups and results of the meta-analysis.

Subgroup	Source	Type of pain	Treatment duration	Heterogeneity test	Study	Events	Sample Size	Prevalence (CI 95%)	Publication Bias
A	General Population Surveys	General Chronic pain	Long-term use	Q = 12.44; df = 1; *p* < 0.001 *I* ^2^ = 91.96 There is heterogeneity	Birke, 2016	63	3,501	1.8 (1.4–2.3)	—
					Fredheim, 2014	417	14,477	2.9 (2.6–3.2)	
					Summary Prevalence			2.3 (1.5–3.6)	
B	General Population Surveys	General Chronic pain	Short-term use	Q = 275.47; df = 4; *p* < 0.001 *I* ^2^ = 98.55 There is heterogeneity	Miller, 2017	393	3,146	12.5 (11.4–13.7)	Egger’s test: *p* = 0.1119 Begg’s test: *p* = 0.2207
					Birke, 2016	137	3,501	3.9 (3.3–4.6)	
					Fredheim, 2014	1787	14,477	12.3 (11.8–12.9)	
					Azevedo, 2013	76	1786	4.3 (3.4–5.3)	
					Kurita, 2012	407	3,305	12.3 (11.2–13.5)	
					Summary Prevalence			8.1 (5.6–11.6)	
C	General Population Surveys	Chronic Low Back Pain	Unspecified	Q = 135.96; df = 1; *p* < 0.001 *I* ^2^ = 99.26 There is heterogeneity	Shmagel. 2018	132	700	18.9 (16.1–21.9)	—
					Gouveia, 2017	24	1,487	1.6 (1.1–2.4)	
					Summary Prevalence			5.8 (0.5–45.5)	
D	Health records or Medical Surveys	General Chronic pain	Unspecified	Q = 901.59; df = 2; *p* < 0.001 *I* ^2^ = 99.78 There is heterogeneity	Lin, 2019	942	2,846	33.1 (31.4–34.9)	Egger’s test: *p* = 0.1662. Begg’s test: *p* = 0.6015
					Romanelli, 2017	69,935	120,481	58.0 (57.8–58.3)	
					Henderson, 2013	356	1,088	32.7 (30.0–35.6)	
					Summary Prevalence			41.0 (23.3–61.3)	
E	Health records or Medical Surveys	Musculoskeletal Conditions	Unspecified	Q = 509.24; df = 4; *p* < 0.001 There is heterogeneity	Callhof, 2019	531	3,564	14.9 (13.8–16.1)	Egger’s test: *p* = 0.2391 Begg’s test: *p* = 0.3272
					Rodoni, 2019	262	499	52.5 (48.1–56.9)	
					Van den driest, 2019	186	842	22.1 (19.4–25.0)	
					Sites, 2018	2,564	19,566	13.1 (12.6–13.6)	
					Knoop, 2017	79	656	12.0 (9.8–14.8)	
					Summary Prevalence			20.5 (12.9–30.9)	
F	Health records or Medical Surveys	Fibromyalgia	Unspecified	Q = 4.412; df = 1; *p* = 0.036 *I* ^2^ = 77.34 There is heterogeneity	Vincent, 2015	249	1,111	22.4 (20.1–25.0)	—
					Häuser, 2012	381	1,465	26.0 (23.8–28.3)	
					Summary Prevalence			24.5 (22.9–26.2)	

All the models are random effects models, given the heterogeneity observed in all the subgroups. Q, Cochran’s Q; df, degrees of freedom; *p*, *p*-value; *I* ^2^, The *I* ^2^ statistic (percentage of variation across studies that is due to heterogeneity)

Six subgroups were established. Group A included studies carried out in the general population including people with CNCP, where the duration of the use of opioids was long-term or persistent (Fredheim et al., 2014; Birke et al., 2016). Group B included studies in the general population including people with CNCP, but in which the duration of the use of opioids was short-term (Kurita et al., 2012; Azevedo et al., 2013; Fredheim et al., 2014; Birke et al., 2016; Miller et al., 2017). Group C included studies in the general population which analyze people with chronic low back pain (CLBP) who had been using opioids (Gouveia et al., 2017; Shmagel et al., 2018). Group D consisted of studies that included patients with CNCP from health registries who had been using opioids (Henderson et al., 2013; Romanelli et al., 2017; Lin et al., 2019). Group E included studies with patients from medical surveys with musculoskeletal conditions [comprising musculoskeletal pain, osteoarthritis and CLBP, following The International Classification of Diseases (Ministerio De Sanidad Consumo y Bienestar Social, 2020)] and who had been using opioids at the moment of the study (Knoop et al., 2017; Sites et al., 2018; Rodondi et al., 2019; Van Den Driest et al., 2019; Callhoff et al., 2020). Finally, group F included studies of fibromyalgia patients from medical surveys who had been using opioids (Häuser et al., 2012; Vincent et al., 2015) (Table 3).

Studies carried out in a population of a specific age (Fain et al., 2017), those that could not be compared with any other study, such as those focused on a specific type of pain (Kingsbury et al., 2014; Marschall et al., 2015), those from specific sample sources (Wand et al., 2016; Scala et al., 2018; Miller et al., 2019), and those focused on visits rather than the patients (with the potential overlapping of the records of the patients) (Larochelle et al., 2015; Ahn et al., 2016) were excluded from the meta-analysis.

The measurement of effect for each study and the summary measure (prevalence of opioid use, defined as the number of subjects taking opioids divided by the number of individuals with CNCP) were calculated with 95% CI. Also, the logit transformation with 95% CI and with standard error and variance were obtained to stabilize the variance (Barendregt et al., 2013). Studies were weighted according to the prevalence of the effect size and the inverse of the study variance.

The heterogeneity between the studies was determined by the DerSimonian and Laird method with Cochran’s Q statistic. As heterogeneity was observed in all the study subgroups, random effects models were performed, which considers the variability of the results due to the differences between the studies. The proportion of total variability due to the heterogeneity of the studies was estimated using the *I* ^2^ value. The results of the meta-analysis are presented in forest plots. To assess the potential publication bias in groups with three or more studies, a funnel plot, along with Begg’s rank correlation and Egger’s weighted regression methods, were used. Two-tailed *p* < 0.05 was considered indicative of a statistically significant publication bias.

Finally, a sensitivity analysis was carried out in groups with three or more studies to determine the influence of each of the studies on the overall estimate of the effect, and therefore the robustness or stability of the final measurement obtained, through influence graphs.

The data were analyzed using Comprehensive Meta-Analysis Software Version 3.0 (Biostat, Englewood, NJ, USA).

## Results

**FIGURE 1:**
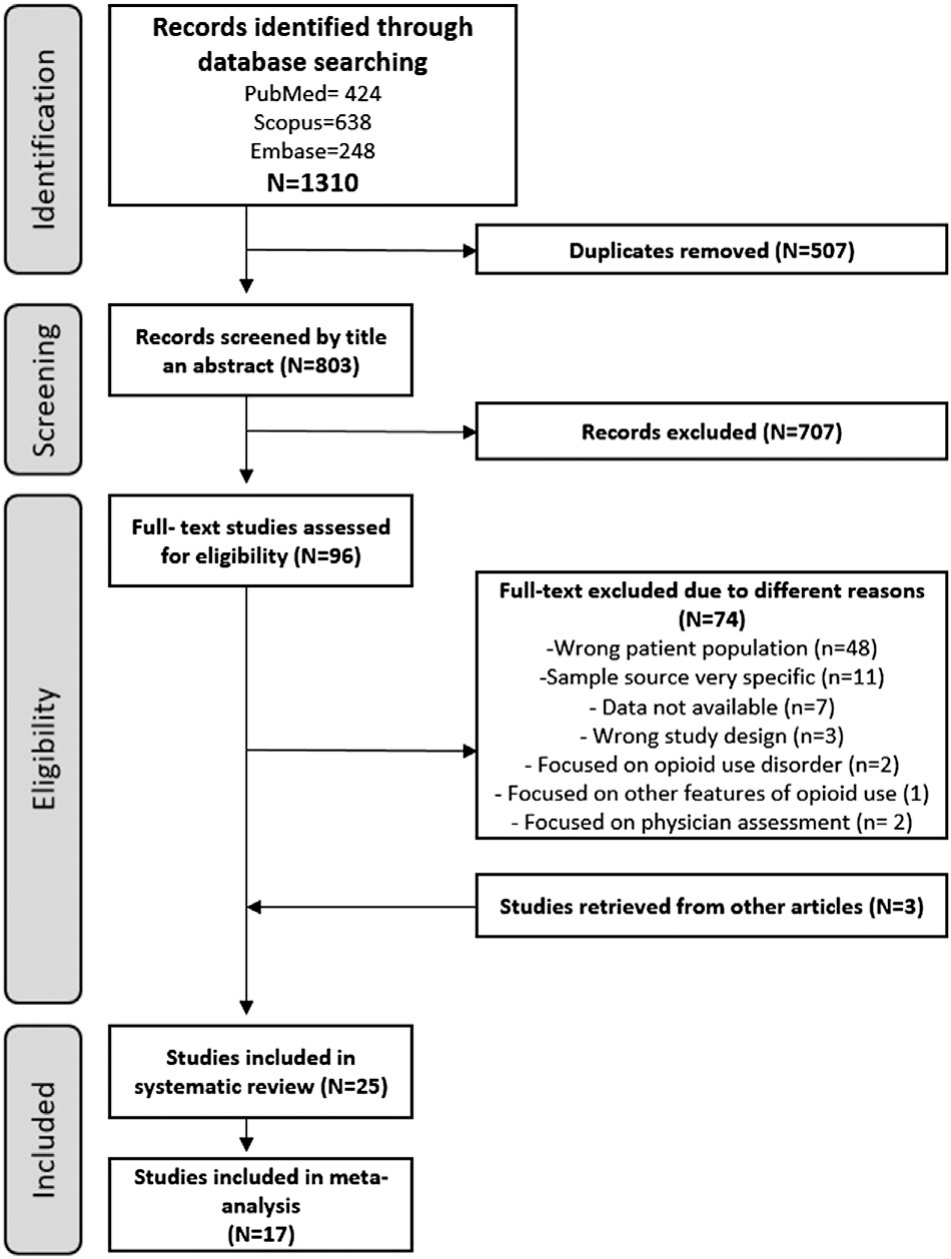
Flowchart.

The search identified 1,310 potential studies. After the selection process (Figure 1), 22 suitable studies were identified. Three more studies obtained by the additional search strategies (citation search) were added.

### Risk of Bias Assessment

Twenty-two of the 25 studies that remained fulfilled at least seven items of the checklist, indicating a low risk of bias. The response rate (Q9) was not reported by seven studies and the use of valid methods for the identification of the condition (Q6) was not reported by 4 (Table 1).

### Study Characteristics

Out of these 25 studies, nine had been performed in the general population (Kurita et al., 2012; Azevedo et al., 2013; Fredheim et al., 2014; Kingsbury et al., 2014; Ahn et al., 2016; Birke et al., 2016; Gouveia et al., 2017; Miller et al., 2017; Shmagel et al., 2018), and sixteen in patients with CNCP from medical surveys or medical records (Häuser et al., 2012; Henderson et al., 2013; Larochelle et al., 2015; Marschall et al., 2015; Vincent et al., 2015; Wand et al., 2016; Fain et al., 2017; Knoop et al., 2017; Romanelli et al., 2017; Scala et al., 2018; Sites et al., 2018; Lin et al., 2019; Miller et al., 2019; Rodondi et al., 2019; Van Den Driest et al., 2019; Callhoff et al., 2020) (Table 2). The data were gathered from thirteen countries. Most of the studies (n = 15) were restricted to adult populations (18 years or older), whereas two study also included adolescents (≥16 years) (Kurita et al., 2012; Birke et al., 2016), three included children (all ages) (Henderson et al., 2013; Ahn et al., 2016; Miller et al., 2017), and one only included people over 65 (Fain et al., 2017). In four studies, the age was not specified. Thirteen studies were performed in patients suffering from a chronic painful process of specific cause (e.g., musculoskeletal pain) (Häuser et al., 2012; Kingsbury et al., 2014; Larochelle et al., 2015; Vincent et al., 2015; Ahn et al., 2016; Wand et al., 2016; Gouveia et al., 2017; Knoop et al., 2017; Shmagel et al., 2018; Sites et al., 2018; Rodondi et al., 2019; Van Den Driest et al., 2019; Callhoff et al., 2020). The reported participation rates in the studies ranged from 37% (Rodondi et al., 2019) to 84.8% (Miller et al., 2017), but in some instances, the information given by the authors was missing or unclear (Table 2). CP was defined as pain lasting at least 6 months in five of the studies included (Kurita et al., 2012; Fredheim et al., 2014; Birke et al., 2016; Miller et al., 2017; Scala et al., 2018), while in the rest, it was considered as pain lasting longer than 3 months. The prevalence of CNCP in the studies carried out in the general population ranged from 6.8% (Romanelli et al., 2017) to 35.7% (Azevedo et al., 2013) (Table 2).

### Prevalence of Opioid use

Out of the nine studies set in the general population, two distinguished between short-term or occasional opioid users and long-term or persistent opioid users (Fredheim et al., 2014; Birke et al., 2016). The prevalence was higher in those in which the use was short or occasional (3.9%–12.3% vs. 1.8%–2.9%) (Kurita et al., 2012; Fredheim et al., 2014; Birke et al., 2016). Three studies (out of nine in the general population) focused on CLBP, and the prevalence ranged from 1.6% (Gouveia et al., 2017) to 18.8% (Shmagel et al., 2018). Another study retrieving data from five countries focused on osteoarthritis, the total prevalence of opioid being use 16.7% (Kingsbury et al., 2014).

In the studies analyzing the population from medical registries or medical surveys, the use of opioids was variable: 32.7% in patients treated in general practices (Henderson et al., 2013), 28.4% among commercially insured patients (Miller et al., 2019) and 64.4% in patients receiving care in a pain center (Scala et al., 2018). In the studies in patients suffering a specific pain condition, the use of opioids ranged from 13.1 to 20.8% in the case of musculoskeletal pain (Larochelle et al., 2015; Sites et al., 2018), from 12% (Knoop et al., 2017) to 22% in osteoarthritis (Callhoff et al., 2020) and from 8.4% (Häuser et al., 2012) to 22.4% in fibromyalgia (Vincent et al., 2015). The highest prevalence of opioid use was 81.2% in a study performed in a nursing home with people ≥65 years (Fain et al., 2017) (Table 2).

### Factors Associated with the use of Opioids

Seven of the studies included in the review analyzed the factors associated with the use of opioids, reporting a greater use of these drugs in men (Fredheim et al., 2014; Romanelli et al., 2017), in young people (Fredheim et al., 2014; Larochelle et al., 2015; Romanelli et al., 2017), in patients receiving prescriptions for different kinds of drugs (Fredheim et al., 2014), in people with a lower educational level (Shmagel et al., 2018), in smokers (Rodondi et al., 2019), and in patients without insurance or with noncommercial insurance, especially Medicaid and Medicare, vs. those with private insurance (Larochelle et al., 2015; Romanelli et al., 2017; Lin et al., 2019) (Table 2).

The use of opioids was also related to the physician. Patients followed by a physician had higher odds of being prescribed an opioid than naive patients (Lin et al., 2019). Moreover, if the primary care physician was trained in complementary medicine, he/she was significantly less likely to prescribe opioids (Rodondi et al., 2019).

Additionally, opioid use was greater in patients with a pain-related disability (Azevedo et al., 2013) and in those with more CP conditions (Romanelli et al., 2017; Shmagel et al., 2018). However, patients with a higher score on the Charlson Comorbidity Index (2–3 vs. 0) had lower odds of receiving an opioid (Romanelli et al., 2017) (Table 2).

Race was related to the use of opioids in two studies, which showed that non-white patients (Larochelle et al., 2015) and Asian patients (Romanelli et al., 2017) were less likely to receive opioids than non-Hispanic white patients (Table 2).

### Results of the Meta-Analysis

**FIGURE 2:**
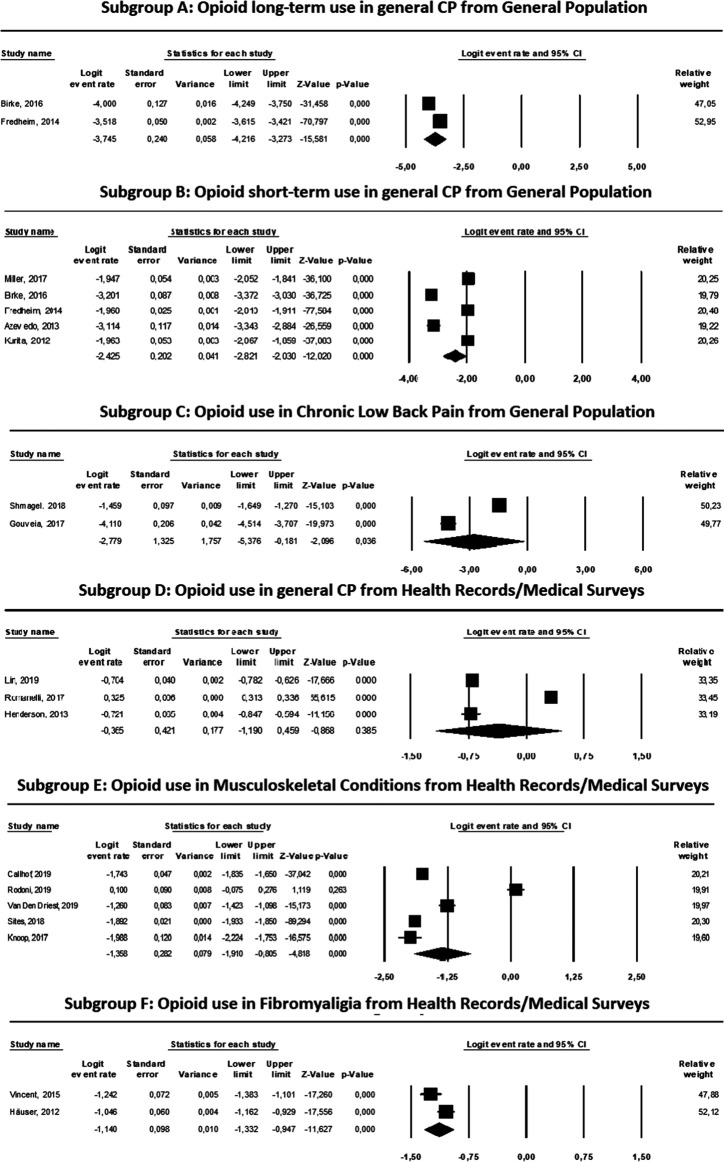
Results and ForestPlots of the meta-analyses for opioid use in different types of pain from diferent sources.

The characteristics and results of the meta-analysis (heterogeneity tests, estimated prevalences with 95% CI, relative weights and tests for publication bias) of the studies included in each of the six subgroups are shown in Table 3, and the results of the logit transformations are shown in Figure 2.

As shown in Table 3, we found heterogeneity within the groups, demonstrating a marked variability among the estimates (*I* ^2^ > 77, *p* < 0.05, in all cases). Therefore, the model used for the estimations of the summarized prevalence and logit transformation was the random effects model.

Among the results obtained, it is noteworthy that in the general population, the prevalence of long-term opioid use among patients with general CNCP was 2.3% (95% CI: 1.5–3.6%), the prevalence of short-term opioid use was 8.1% (95% CI: 5.6–11.6%), and the prevalence in CLBP was 5.8% (95% CI: 0.5–45.5%). The prevalence among patients from health registries or medical surveys was 41% (95% CI: 23.3–61.3%) in patients with general CNCP. The prevalence in patients with musculoskeletal conditions was 20.5% (95% CI: 12.9–30.9%) and 24.5% in patients with fibromyalgia (95% CI: 22.9–26.2%) (Table 3).

Regarding the results obtained with the logit transformations, negative summary measures were obtained in all the groups, ranging from −3.745 in subgroup A, comprising subjects receiving long-term opioid treatment for general CNCP from general population surveys, to −0.365 in subgroup D, patients receiving opioids for general CNCP from health records or medical surveys (Figure 2).

**FIGURE 3:**
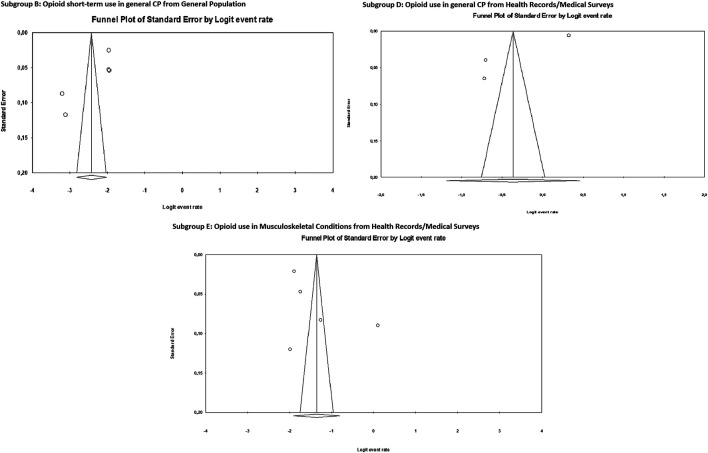
Publication bias. Funnel plots of the subgroups B, D and E of the meta-analyses.

Figure 3 shows the funnel plot for the meta-analysis of subgroups B, D and E, suggesting no evidence of publication bias. Neither Egger’s test nor Begg’s test were statistically significant for the publication bias (Table 3).

**FIGURE 4:**
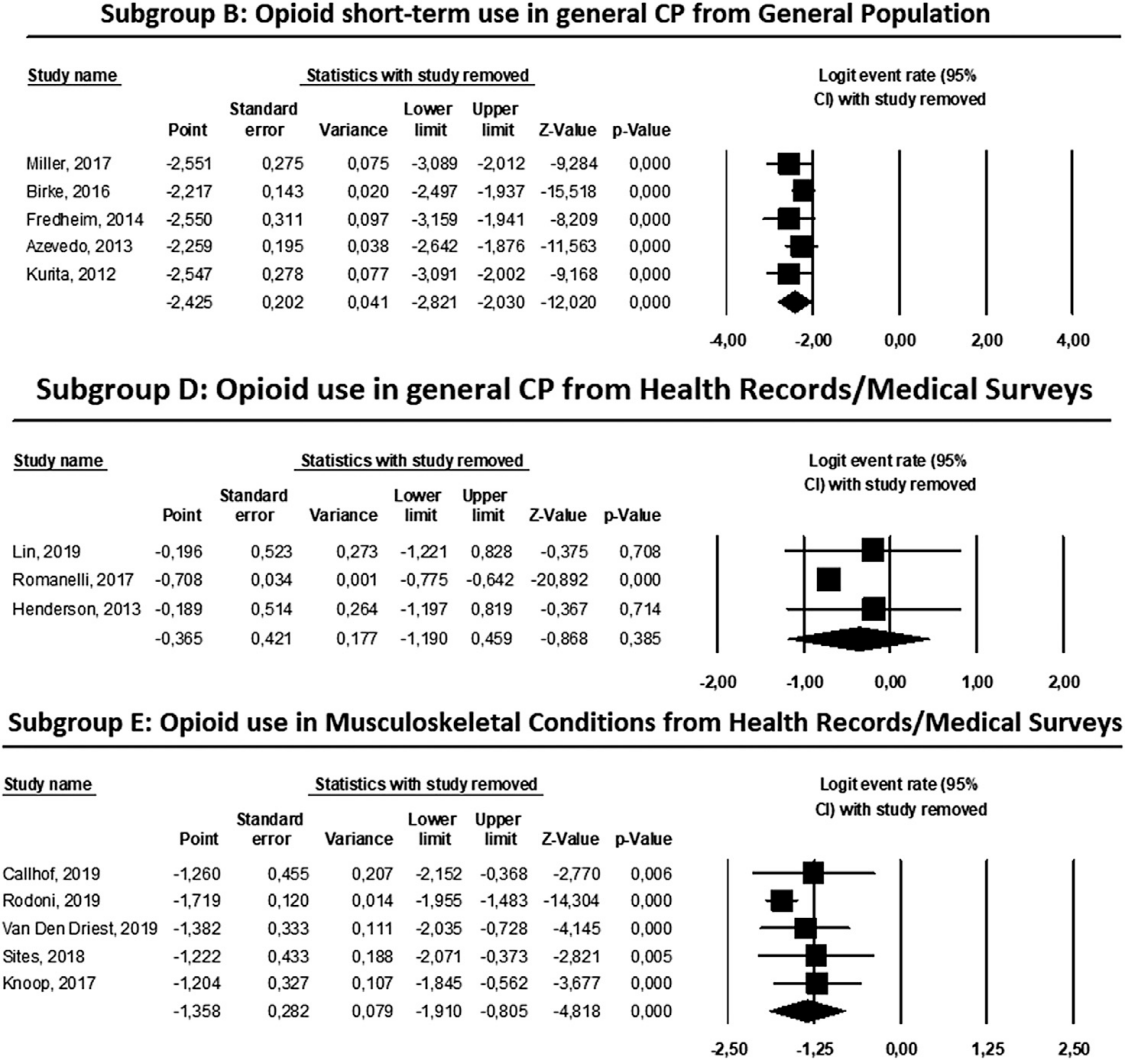
Influence graphics for sensibility analysis of the subgroups B, D and E of the meta-analyses.

Finally, Figure 4 shows the result of the sensitivity analysis for subgroups B, D and E, indicating in the three cases that none of the studies included would substantially change the overall result of the summarized logit transformation if the studies were eliminated from the meta-analysis. This finding indicates that the results are robust, since none of the studies exerted a great influence on the final result.

## Discussion

This study analyzes the information published about the prevalence of the use of opioids in patients with CNCP and examines the factors associated with their use.

The results reveal that there were differences in the prevalence of the use of these drugs depending on the length of the treatment (2.3% in long duration or 8.1% in occasional use) (Fredheim et al., 2014; Birke et al., 2016). It was also observed that when the information derives from health registries, the prevalence is much higher than in the general population, and more variable depending on the chronic pain condition.

The lower prevalence found in patients with longer treatments seems reasonable if we take into account, on the one hand, the prescribers’ concern about the risk of addiction and the improper use of these drugs by some patients (Allen et al., 2013) and, on the other hand, the treatment dropout, possibly due to the appearance of analgesic tolerance, induced hyperalgesia, side effects frequently associated with these drugs, and insufficient pain relief (Kalso et al., 2004; Noble et al., 2008; Sehgal et al., 2013). In this line, a recent systematic review about opioids for CNCP concludes that the benefits of opioids for pain and functioning are similar to non-opioid alternatives. Opioid use was associated with small improvements in pain and physical functioning, and increased risk of vomiting compared with placebo (Busse et al., 2018).

The higher prevalence in the studies based on health registries could be explained because these patients are usually treated in specialized pain units, especially those patients with complex types of pain conditions who do not respond to conventional treatment and require special drugs, such as opioids (Henderson et al., 2013; Pérez et al., 2013; De Sola et al., 2020). Furthermore, a lack of healthcare providers offering effective treatment alternatives is likely to have an impact on other treatment choices (White et al., 2019). Another possible reason is the difficulty in opioid deprescribing when patients have poor function and unremitting pain or aberrant behavior and misuse, since tapering is a complex process with little current guidance available (White et al., 2019).

Regarding the specific pathologies, the prevalence in patients with musculoskeletal conditions was 20.5% and in patients with fibromyalgia 24.5% (Luo et al., 2004; Romanelli et al., 2017). Musculoskeletal conditions are one of the most common causes of pain (Breivik et al., 2006; Pierce et al., 2019) and the use of weak opioids was recommended to relieve pain and disability in the short-term in these patients (Airaksinen et al., 2006). However, recent studies have found that LTOT is associated with unrelieved pain, greater functional impairment, and lower return to work rates (Turner et al., 2016; Krebs et al., 2018). In line with this, current treatment guidelines do not recommend opioids for fibromyalgia management (Peng et al., 2015), possibly because opioids are unable to target the pathophysiological processes involved in this central sensitization syndrome (Menzies et al., 2017). Our findings suggest that, despite a lack of scientific support of opioid treatment in people with fibromyalgia and for musculoskeletal conditions, clinicians are nevertheless prescribing them for symptom management in these patients.

In the analysis of the factors associated with the use of opioids, younger individuals showed greater use. One explanation could be that opioids are not always recommended for the elderly population due to a higher probability of liver or kidney dysfunction, greater risk of respiratory depression, drug interactions, organ dysfunction, co-morbidity and side effects, such as constipation, drowsiness or sedation, which can have more serious consequences in this population (Romanelli et al., 2017). In addition, it has been shown that medical personnel sometimes underestimate pain in the elderly, which leads to a lower prescription of opioids in these patients (Raymond and John, 2002; Seers et al., 2018).

Regarding race, different studies have shown that the pain experience is different according to the ethnic group. This finding has been attributed to different responses to painful stimuli and the different coping strategies for managing pain between groups (Campbell and Edwards, 2012; Larochelle et al., 2015; Ringwalt et al., 2015; Romanelli et al., 2017). Additionally, according to Anderson et al. (2009), there are other factors that could influence these differences, such as selective care and differences in the process of evaluation and allocation of treatment according to the ethnic group of the patient.

Another factor to consider is the type of care received by the patient. The type of medical insurance can influence the manner of approaching the pain and consequently determine the use of opioids. Patients with private insurance have been reported to obtain better results than patients with public coverage (Meineche-Schmidt et al., 2012) since, in addition to the fact that the care is more immediate, a multidisciplinary approach is more common and produces better results, decreasing the use of analgesic treatment (Becker et al., 2000). In this vein, Rodondi et al. (2019) highlighted that the training of the physician in complementary medicine also results in the prescription of less opioid treatments, since being specialized in integrative and complementary medicine could help physicians to inform and guide patients about the most effective treatment options, their potential interactions with conventional therapies, and their side effects.

Finally, it would be reasonable to think that in those studies where the prevalence of CP is higher, the use of opioids would also be greater (Barnett et al., 2012). However, when we compare the results from different countries, this hypothesis is not confirmed, as the factors that may be important are the method of data collection and the characteristics of the population included in the studies (Miller et al., 2017; Steingrímsdóttir et al., 2017). Likewise, the cultural perceptions of pain could help us to understand the differences in the prevalence of opioid use, the perception of opioid use, the widespread marketing campaign for opioids, and the regulations controlling the prescription of opioids (Severino et al., 2018). Most of the studies included in this review were performed in the US and Western Europe, where there are significant differences in healthcare systems and healthcare regulatory oversight, the financial incentive behind the treatment of pain, and restrictions on the length of validity for these drugs, among others (Meyer et al., 2020).

Some limitations of this review should be noted. It is worth mentioning that three of the subgroups in the meta-analysis included only two studies. The minimum number of studies to include in a meta-analysis has been discussed previously in the literature, without clear agreement (Cooper et al., 2009; Valentine et al., 2010; Pigott, 2012). Some researchers consider that a minimum of five studies are desirable, or even required. Others argue that, as long as the studies meet the quality criteria and statistical requirements, the meta-analysis can be carried out, as it is just a statistical combination of the results. The number of studies in the literature on a topic is out of our control, and the lack of studies on these topics (in our case, studies carried out in the general population focused on the prevalence of the long-term use of opioids; in the general population focused on the prevalence of the use of opioids in CLBP; and from health records or medical surveys focused on the use of opioids in fibromyalgia) is itself a relevant result, and it shows the need for further research on the topics. Of course, the number of studies has a direct impact on the statistical power and precision, but if those few studies are relevant and their quality is high, we believe that it is worth drawing conclusions from them. In this vein, Pigott (2012) argued that the quick answer for the minimum number of studies is two, but recommend computing the statistical power a priori “using assumptions about the size of an important effect in a given context, and the typical sample sizes used in a given field.” Finally, Valentine et al. (2010) state that a meta-analysis is always the best option to synthesize information (even if we have few studies) as other alternatives “are likely to be based on less defensible assumptions and on less transparent processes.” Consequently, we decided to perform these three meta-analyses which, however, need to be interpreted with caution, given the limited statistical power.

In order to strengthen our findings, we attempted to limit the impact of the clinical and methodological heterogeneity, classifying the studies into subgroups with similar characteristics. However, the tests for statistical heterogeneity among the studies included in the meta-analyses still demonstrated substantial variability between them (*I* ^2^ > 77, *p* < 0.05). A stratified analysis grouped by the different nationalities of the patients or by the different definitions of the prevalence of use of opioids in each study could have been another potential way of classification based on the importance of cultural aspects, healthcare systems, and healthcare regulations in opioid use. However, the few studies with this information available, and the marked heterogeneity within the groups made another way to stratify them impossible. Also, the differences in sociodemographic structure, socioeconomic level and the duration of pain could contribute to the differences between the study populations. However, without access to individual patient data, it was not possible to control these factors.

Another limitation of our review was the risk of bias in the studies. Although 22 out of the 25 studies fulfilled at least seven items of the checklist, only 12 fulfilled the nine items of the Critical Appraisal Checklist for Studies Reporting Prevalence Data, and just over half of the 25 studies included were not primarily designed to produce prevalence data (Table 2). This was reflected in the variability of reporting of important variables. For example, population denominators and response rates were not always identifiable and there were occasional numerical discrepancies between the data presented in the study abstract, main text, and results tables. Despite the fact that some of the studies could have a potential bias, the flaws are not sufficient to invalidate the results since they satisfied other criteria in the assessment of the risk of bias and provided important information in line with the objectives of this review.

Despite extensive database searches, it is possible that some references from the gray literature were missed. Furthermore, language is one of the methodological limitations. All the studies were in English, conducted in predominantly Western settings, making generalizability to other parts of the world difficult. As a strength of the study, we would like to highlight its novelty since, to the best of our knowledge, no systematic review and meta-analysis of the prevalence of the therapeutic use of opioids has been published previously.

## Conclusion

This study shows that the prevalence of opioid use among patients with CNCP was higher in clinical studies based on health registries and in subjects with short or occasional use compared to those with long term use. Men, younger people, more CP conditions, and patients without insurance or with noncommercial insurance were most related to opioid use. In contrast, non-white and Asian patients, and those treated by a physician trained in complementary medicine were less likely to use opioids.

## Author Contributions

I Failde supervised the study. HS and MD led the writing. HS, MD, AS and PJ conducted the search strategy and the analysis. All the authors conceptualized the study, contributed to its writing, and made a critical review of the article.

## Conflict of Interest

The authors declare that the research was conducted in the absence of any commercial or financial relationships that could be construed as a potential conflict of interest.
