# Supplementary material for: Prevalence of Therapeutic use of Opioids in Chronic non-Cancer Pain Patients and Associated Factors: A Systematic Review and Meta-Analysis
Source: Front Pharmacol. 2020 Nov 18;11:564412. doi: 10.3389/fphar.2020.564412 (PMC7750787; doi:10.3389/fphar.2020.564412)
Supplement: Supplementary file 2 [file datasheet2.docx]

**SUPPLEMENTARY MATERIAL II**

**Table 1**. Combined search terms used for this systematic review in each database, number of studies found, and search term used for the purpose of Figure 1.

| **Systematic review Medline-PubMed (N=424)** |
| --- |
| opioid*[Title/Abstract] AND analgesic* AND pain[Title/Abstract] AND "cross-sectional" AND ("last 10 years"[PDat] AND Humans[Mesh]) |
| **Systematic review Scopus (N=638)** |
| ( ( ( TITLE-ABS-KEY ( opioid* )  AND  ALL ( analgesic* )  AND  TITLE-ABS-KEY ( pain )  AND  TITLE-ABS-KEY ( "Cross-Sectional" ) ) ) )  AND  ( LIMIT-TO ( DOCTYPE ,  "ar" ) )  AND  ( LIMIT-TO ( PUBYEAR ,  2019 )  OR  LIMIT-TO ( PUBYEAR ,  2018 )  OR  LIMIT-TO ( PUBYEAR ,  2017 )  OR  LIMIT-TO ( PUBYEAR ,  2016 )  OR  LIMIT-TO ( PUBYEAR ,  2015 )  OR  LIMIT-TO ( PUBYEAR ,  2014 )  OR  LIMIT-TO ( PUBYEAR ,  2013 )  OR  LIMIT-TO ( PUBYEAR ,  2012 )  OR  LIMIT-TO ( PUBYEAR ,  2011 )  OR  LIMIT-TO ( PUBYEAR ,  2010 )  OR  LIMIT-TO ( PUBYEAR ,  2009 ) )  AND  ( LIMIT-TO ( LANGUAGE ,  "English" )  OR  LIMIT-TO ( LANGUAGE ,  "Spanish" ) )  AND  ( LIMIT-TO ( EXACTKEYWORD ,  "Human" ) ) |
| **Systematic review Embase (N=248)** |
| opioid*:ab,ti AND analgesic*:ab,ti AND pain:ab,ti AND 'cross-sectional study' AND [2009-2019]/py AND 'human'/de |

**Table 2.** Reasons for the exclusion of the studies not included in the systematic review and meta-analysis (N=74).

| **Author, year** | **Reason for exclusion** |
| --- | --- |
| Austin et al, 2017 | Wrong patient population |
| Bastian et al, 2017 | Wrong patient population |
| Blanco et al, 2012 | Wrong patient population |
| Boehnke et al, 2016 | Sample source very specific |
| Buse et al, 2012 | Wrong patient population |
| Carriere et al, 2017 | Focused on other features of opioid use |
| Carriere et al, 2018 | Wrong patient population |
| Challa et al. 2017 | Sample source very specific |
| Chan et al, 2018 | Sample source very specific |
| Chang et al, 2014 | Wrong patient population |
| Civardi et al, 2018 | Wrong study design |
| Darnall et al, 2011 | Sample source very specific |
| Daubresse et al, 2013 | Wrong patient population |
| Desai et al, 2019 | Wrong patient population |
| Deyo et al, 2013 | Sample source very specific |
| Elsesser et al, 2017 | Wrong patient population |
| Enthoven et al, 2014 | Wrong study design |
| Erdeljić et al, 2011 | Sample source very specific |
| Feinberg et al, 2018 | Sample source very specific |
| Fischer et al, 2010 | Focused on opioid use disorder |
| Fleckenstein et al, 2010 | Focused on physician assessment |
| Fredheim et al, 2011 | Focused on opioid use disorder |
| Frenk et al, 2019 | Wrong patient population |
| Gadzhanova et al, 2015 | Sample source very specific |
| Gomes et al, 2011 | Wrong patient population |
| Guite et al, 2018 | Wrong patient population |
| Hansen et al, 2015 | Wrong patient population |
| Harle et al, 2014 | Wrong patient population |
| Hauser et al, 2018 | Wrong patient population |
| Healey et al, 2018 | Data not available |
| Hemmingsson et al, 2018 | Wrong patient population |
| Holliday et al, 2013 | Focused on physician assessment |
| Hoppe et al, 2015 | Wrong patient population |
| Jobski et al, 2017 | Wrong patient population |
| Kozma et al, 2014 | Wrong patient population |
| Lin et al, 2017 | Wrong patient population |
| Lin et al, 2018 | Wrong patient population |
| Mailis-Gagnon et al, 2011 | Wrong patient population |
| Marcum et al, 2011 | Wrong patient population |
| Martel et al, 2019 | Sample source very specific |
| Miller et al, 2018 | Sample source very specific |
| Montero et al, 2012 | Data not available |
| Montero Matamala et al, 2011 | Wrong patient population |
| Moreira de Barros et al, 2019 | Wrong patient population |
| Muller et al, 2019 | Wrong patient population |
| Narayana et al, 2015 | Wrong patient population |
| O’Gara et al, 2016 | Wrong patient population |
| Pérez et al, 2009 | Wrong patient population |
| Pérez et al, 2013 | Data not available |
| Pierce et al, 2019 | Wrong patient population |
| Pitkala et al, 2015 | Wrong patient population |
| Pokela et al, 2010 | Wrong patient population |
| Rasu et al, 2016 | Data not available |
| Rasu et al, 2018 | Wrong patient population |
| Rasu et al, 2013 | Wrong patient population |
| Rivera et al, 2016 | Wrong study design |
| Roxburgh et al, 2011 | Wrong patient population |
| Ruscitto et al, 2015 | Wrong patient population |
| Samison et al, 2017 | Wrong patient population |
| Samuelsen et al, 2016 | Data not available |
| Sawyer et al, 2010 | Wrong patient population |
| Sites et al, 2014 | Wrong patient population |
| Steinman et al, 2015 | Wrong patient population |
| Stokes et al, 2019 | Wrong patient population |
| Stompór et al, 2019 | Sample source very specific |
| Taylor-Stokes 2011 | Data not available |
| Thomas et al, 2015 | Wrong patient population |
| Todd et al, 2018 | Wrong patient population |
| Veal et al, 2015 | Wrong patient population |
| Westergaard et al, 2015 | Wrong patient population |
| Yackey et al, 2018 | Wrong patient population |
| Zheng et al, 2017 | Data not available |
| Zin et al, 2014 | Wrong patient population |
| Zin et al, 2017 | Wrong patient population |
